# Book-Sharing for Toddlers with Clefts (BOOST): Protocol for a randomized controlled trial of a remote intervention to promote language development in children with cleft palate

**DOI:** 10.1371/journal.pone.0304630

**Published:** 2024-06-13

**Authors:** Brent R. Collett, Emily R. Gallagher, Alexis L. Johns, Cindy O. Trevino, Brian G. Leroux, Frederick Shic, Canice E. Crerand, Adriane L. Baylis, Caitlin A. Cummings, Lupita Santillan

**Affiliations:** 1 Center for Child Health, Behavior, and Development, Seattle Children’s Research Institute, Seattle, Washington, United States of America; 2 Department of Psychiatry and Behavioral Sciences, University of Washington School of Medicine, Seattle, Washington, United States of America; 3 Seattle Children’s Craniofacial Center, Seattle Children’s Hospital, Seattle, Washington, United States of America; 4 Department of Pediatrics, University of Washington School of Medicine, Seattle, Washington, United States of America; 5 Keck School of Medicine, University of Southern California, Los Angeles, California, United States of America; 6 Division of Plastic and Maxillofacial Surgery, Children’s Hospital Los Angeles, Los Angeles, California, United States of America; 7 Department of Biostatistics, University of Washington, Seattle, Washington, United States of America; 8 Department of Oral Health Sciences, University of Washington, Seattle, Washington, United States of America; 9 Department of Pediatrics, The Ohio State University College of Medicine, Columbus, Ohio, United States of America; 10 Department of Plastic and Reconstructive Surgery, The Ohio State University College of Medicine, Columbus, Ohio, United States of America; 11 Center for Biobehavioral Health, Nationwide Children’s Hospital, Columbus, Ohio, United States of America; 12 Department of Plastic and Reconstructive Surgery, Nationwide Children’s Hospital, Columbus, Ohio, United States of America; 13 Department of Speech Pathology, Nationwide Children’s Hospital, Columbus, Ohio, United States of America; Public Library of Science, UNITED KINGDOM

## Abstract

**Background:**

Children with cleft palate, with or without cleft lip (CP±L), exhibit language delays on average compared to children without clefts. Interventions to address these disparities are scarce. In this multi-center study, Book Sharing for Toddlers with Clefts (BOOST), we will test a remote, parent-focused intervention to promote language development in children with CP±L.

**Objectives:**

The study will test two primary hypotheses. First, toddlers randomized to BOOST will exhibit better language outcomes than children receiving standard-of-care (SOC). Second, we hypothesize that the BOOST program’s effect on language outcomes is mediated by the frequency and quality of parent-child reading interactions.

**Methods:**

The study is a randomized-controlled trial comparing the BOOST group to a SOC comparison group. We will enroll N = 320 English and/or Spanish-speaking children ages 24–32 months with isolated CP±L (n = 160 per group). Both groups will receive children’s books, and parents will record and upload videos of themselves reading the books with their children using a smartphone app developed for the study. Parents will also complete surveys asking whether they read to their children on five randomly selected days each week. In addition, the BOOST group will participate in 3 remote dialogic book-sharing intervention sessions via Zoom. We will code book-sharing videos to assess parents’ target skill usage and children’s expressive language. End-of-study assessments will include measures of child language outcomes (e.g., clinician-administered measures, parent reports, and naturalistic child language samples).

**Results:**

Enrollment began in April 2024 and will continue through approximately April 2028.

**Conclusion:**

The BOOST study will address a critical gap in the literature on interventions to improve language in children with CP±L. The results will inform the care for toddlers with oral clefts and have potential applications for other populations.

## Introduction

Isolated cleft palate, with or without cleft lip (CP±L), affects approximately 1 in 700 births in the United States [1]. Individuals with CP±L have long been reported to have developmental and learning deficits, particularly in language-mediated skills (e.g., reading). A systematic review of this literature identified 31 studies representing over 10,000 participants [2]. Results showed neurodevelopmental and learning deficits from early childhood through early adulthood. For example, children with clefts score lower than unaffected children on measures of early reading and related pre-reading skills [3–5]. Children and adolescents score lower than their classmates on standardized measures of educational achievement and are more likely to be referred for special education services [6–9]. Overall levels of educational attainment are lower for late adolescents and young adults with clefts than unaffected individuals [8]. A significant limitation common to most of these observational studies has been the inclusion of ethnically and racially homogeneous (i.e., white and non-Hispanic) samples and the exclusion of participants who prefer languages other than English. There is some indication that academic disparities may be greater in Hispanic children with CP±L, though this has not been extensively studied [9].

Hypothesized etiologies for learning problems in individuals with clefts have focused on underlying neurobiological differences (e.g., differences in brain structure or function), detrimental effects of early speech impairment, fluctuating hearing levels, and differential treatment by parents or teachers [2]. Studies testing the efficacy of interventions to offset disparities in early language and learning in this population are scarce. However, Scherer and colleagues have investigated a parent training model to facilitate speech and language development in children with clefts. A randomized study of 30 parent-child dyads (n = 15 treatment, n = 15 control) showed that this approach improved speech articulation for children in the treatment group [10]. Similar results, as well as improvements in expressive and receptive language, were observed in a Brazilian cohort (N = 24) [11] and a recent pilot study examining remote delivery of the intervention (N = 4) [12]. Although promising, the small sample sizes in these studies raise questions about generalizability and the potential to deliver these programs at scale.

In the non-cleft literature, several promising intervention programs exist for children at risk of poor language and academic outcomes. For example, parent-focused interventions to increase the frequency and quality of shared reading have improved children’s language and literacy skills. Dialogic book-sharing (DBS) is one example, emphasizing children’s active engagement in reading by using a child-directed approach, reflecting and encouraging child verbalizations, and encouraging conversational turns [13]. Because DBS emphasizes child language versus text (e.g., the books used often have few words and instead tell stories through illustration), it is appropriate for caregivers with a wide range of literacy [13–16]. Meta-analytic studies suggest that DBS programs lead to robust improvements in parents’ reading behaviors (d ~ 1.0) and moderate improvements in child language outcomes (d ~0.4) [13]. Though the format of these programs varies (e.g., 1:1 meetings with parents, group intervention), benefits have been observed with low-intensity, low-duration interventions (e.g., 90 minutes or more of direct intervention with caregivers). Most of these studies have been conducted with children who are typically developing. However, some evidence supports the efficacy of similar approaches with children who have language delays or are at risk for language delays [14]. Benefits are observed regardless of families’ socioeconomic status (SES), race and ethnicity, and language(s) spoken in the home [15].

Book Sharing for Toddlers with Clefts (BOOST) will be the first large study to examine the efficacy of an intervention to improve early language skills for children with CP±L. The current standard-of-care (SOC) for children with clefts includes developmental monitoring, assessment, and treatment of speech-language concerns. Based on research in non-cleft samples, there is good evidence that parent-focused programs can improve the frequency and quality of shared reading [13–16]. These programs have downstream effects on children’s language (particularly expressive and receptive language), which, in turn, predicts reading acquisition. We have focused our intervention on DBS models to promote frequent interactive parent-child reading.

The BOOST program was developed for remote delivery to accommodate geographically dispersed families. We will meet with families for three 50-minute treatment sessions via Zoom to discuss the application of DBS strategies. Parents will record and share videos of themselves reading with their children between visits. These videos will be used for the intervention (e.g., to identify and reinforce caregiver strengths) and to assess adherence and changes in parents’ reading behaviors. Parents will also complete surveys to evaluate the frequency of shared reading. We will compare children in the BOOST group to children in a group that will receive SOC plus the same books shared with participants in the treatment group. Parents in the SOC group will be asked to upload videos of shared reading and complete shared reading frequency surveys. These procedures will help control the effects of having access to age-appropriate children’s books and being observed.

We hypothesize that (1a) children in the BOOST group will exhibit better expressive and receptive language than children in the SOC group at an end-of-study assessment; (1b) children in the BOOST group will exhibit better language than children in the SOC group during shared reading interactions (e.g., more utterances, more unique words used, larger mean length of utterance); and (2) differences in child language outcomes will be mediated by increased frequency of book-sharing and improvements in the quality of parent-child book-sharing interactions. The study will use a multi-center, parallel-group, randomized controlled trial (RCT) design. We will randomize half of the participants to the BOOST group and half to the SOC comparison group. Parents’ use of target skills will be observed and coded throughout the intervention period, and children’s language outcomes will be assessed at least eight weeks after the end of treatment. Staff involved in observational coding and child outcomes assessment will be unaware of children’s randomization.

## Materials and methods

### Study setting

We have three participating study sites in Washington (WA), California (CA), and Ohio (OH), all with active cleft and craniofacial teams in tertiary-care hospital settings. We seek to enroll a sample that is representative of the demographic characteristics of our patient populations. For example, 37% to 60% of the patients seen in our centers receive Medicaid funding for their healthcare. Approximately 18% of our patients are Latino/Hispanic, and 27% are non-white. After English, Spanish is the most common language spoken by families. Therefore, we translated study materials and ensured that our team included bilingual/Spanish-speaking staff to facilitate the inclusion of Latino/Hispanic families. Translation procedures included initial translation by an outside service and independent back-translation by a separate translator who had not seen the original English versions. Bilingual/Spanish-speaking investigators from our team and content experts then reviewed all translated materials. We made final modifications to ensure the original meaning was retained and that translations were appropriate for a broad Spanish-speaking population. In some instances, we revised the wording of English materials to ensure comprehension and consistency across languages.

### Eligibility criteria

Patients with CP±L ages 24- to 32-months will be eligible if their parent consents to participate and will be available for the duration of the study. We will include participants who received palate surgery at least six months before enrollment, primarily speak English, Spanish, or both languages in the home, and have access to a smartphone or similar device (e.g., tablet) capable of recording and sharing video. Exclusions are diagnosed conditions that affect neurodevelopment (e.g., 22q11.2 deletion syndrome), profound sensory impairment (e.g., blindness, deafness), delivery < 32 weeks’ gestation, placement in foster care or state custody, or a diagnosis of autism spectrum disorder (ASD). Questions related to eligibility will be reviewed by one of the co-investigators (EG) and principal investigator (BC), and eligibility decisions will be documented to ensure consistency.

Staff from each site will complete an initial screening with parents to verify eligibility and obtain informed consent. Once parents consent to participate, baseline measures will be completed, including a demographic and clinical interview and parent-report measures (child expressive language, shared reading frequency). Parents will use their smartphones to record a video of themselves reading with their child and complete a speech task in which the child names a series of pictures. To ensure that families are able and willing to complete study procedures, we will only randomize participants if they complete at least one shared reading survey and upload at least one parent-child reading video at baseline. Parents will use a free app or online website to assess the need for broadband or cellular connectivity support to determine upload and download speeds. As needed, we will assist families with connectivity resources (e.g., a "hotspot") to ensure access to the intervention.

All participants will receive an end-of-study assessment. This will include any participants found to be ineligible after enrollment (e.g., those with new diagnoses) and participants who are not adherent to the intervention protocol or assessments.

### Intervention

All participants will receive five age-appropriate children’s books that they will be encouraged to read with their children. We will ask participants in both groups to video record themselves reading these books with their children and complete surveys of shared reading frequency. Families will use a study app to securely upload book-sharing videos with the research team. This will help to control for possible effects related to being observed. Children will continue to receive SOC treatment, including speech-language therapy and developmental interventions at enrollment or initiated during the study, and follow up with their craniofacial providers.

Participants randomized to the BOOST intervention group will receive support and encouragement to promote shared reading and dialogic book-sharing skills. During the study protocol development phase, we pilot-tested the BOOST program with 40 families recruited from participating centers and made minor modifications based on parent and interventionist feedback. The program is delivered in three 50-minute live, face-to-face telemedicine (Zoom) visits scheduled every 2–3 weeks. The BOOST program encourages parents to increase the frequency of shared reading, introduces skills to promote child engagement and involvement in reading, and offers strategies to promote language development. Depending on parents’ preference, we will provide English or Spanish teaching materials for each of the key skills taught. Interventionists will review the book-sharing videos parents upload between sessions to identify parent strengths, application of target skills, and opportunities for improvement.

#### Training and fidelity

We will hire and train intervention staff with (1) an advanced degree (i.e., master’s degree or higher) in psychology, speech-language pathology, education, or related fields and (2) prior experience working with young children and their parents. We will ensure that our team includes bilingual interventionists.

Interventionist training will be led by one of the study investigators (CT). Training will include core readings [13, 16–23], a review of example intervention sessions, and a review and coding of example parent-child reading interactions. After completing these trainings, interventionists will deliver the program with at least one practice participant in the target age range. Practice sessions will be video-recorded and coded for fidelity. We have adapted an observational measure of treatment fidelity used in earlier parent-focused interventions (COACH-Q) [22, 23]. During pilot testing, we revised the items to suit the BOOST intervention and established operational definitions for coding. The resulting measure includes a checklist of required session components and ratings of interventionist effectiveness coded as 0 = needs improvement, 1 = acceptable, and 2 = excellent. Effectiveness items relate to interventionists’ conceptual accuracy, responsiveness to parents’ needs, active structuring of the session, the teaching of concepts, use of example videos and handouts, strategies to instill hope and foster growth, and overall quality. Ratings of "acceptable" or better and completion of all session elements will be required for interventionists to proceed to deliver the intervention with study participants. Intervention staff will complete additional practice sessions to meet certification requirements when needed.

We will record all intervention sessions to assess treatment fidelity. We will code each interventionist’s first ten visits, with feedback and re-training provided if the average COACH-Q rating is below the "acceptable" range or if any session elements are omitted. We will additionally code a randomly selected 20% of intervention sessions throughout the study. We will lead regular interventionist meetings to review videos and discuss implementation issues.

#### Participant adherence

Participants’ session attendance will be used to assess their adherence to the BOOST program. In addition, to evaluate parents’ engagement in intervention sessions, we have added items related to parent engagement to the COACH-Q observational coding (e.g., whether parents are attentive, ask questions, and provide examples during the visits).

### Study assessments

After enrollment, we will complete a semi-structured caregiver interview to assess family demographics, including race and ethnicity, languages spoken in the home, parent education, parent occupations, maternal age at delivery, parent marital status, family income, and number of children and adults in the home. We will also obtain parents’ reports of their child’s history of developmental interventions, including speech and language therapy, physical therapy, occupational therapy, hearing services, and participation in birth-to-three intervention services. We will obtain parents’ permission to abstract information from children’s medical records, including gestational age at birth, birth complications, cleft diagnosis, age at cleft diagnosis, results from hearing evaluations, age at cleft lip surgery (when applicable), age at cleft palate surgery, other surgical procedures (e.g., tympanostomy tube placement), findings from speech assessments, and frequency of craniofacial clinic visits.

**Fig 1:**
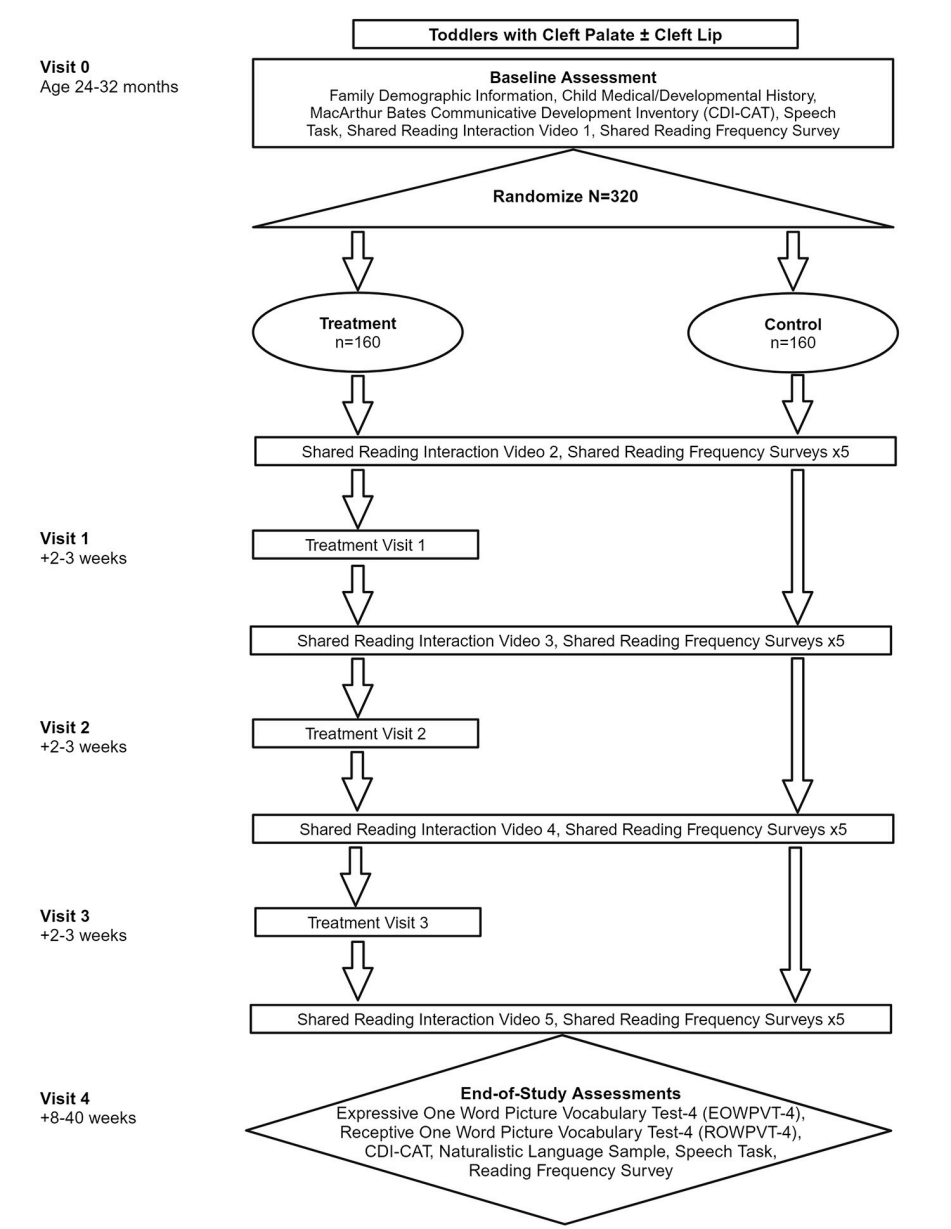
Flow diagram with BOOST study procedures and timeline.

End-of-study outcomes will be assessed at least eight weeks after the intervention period (i.e., after the final parent-child reading video is uploaded). Fig 1 provides a schematic of study procedures, and Table 1 provides a sample participant schedule. All participants will have the opportunity to receive an end-of-study assessment, including any found to be ineligible after enrollment (e.g., those with new diagnoses) and participants who are not adherent to the intervention protocol or assessments.

**Table 1:** Participant timeline.

	Week										
	0	1	2	3	4	5	6	7	8		16–40
**Visit 0: Baseline**	X										
Shared Reading Video 1	X										
Video Recorded Speech Task	X										
Shared Reading Frequency Survey	X										
Family Demographic Information	X										
Child Medical/Developmental History Interview/Chart Abstraction	X										
MacArthur Bates Communicative Development Inventory CAT	X										
**Randomization**	X										
Shared Reading Frequency Surveys (5x per week, randomly selected days)		X	X	X	X	X	X	X	X		
Shared Reading Video 2			X								
**Visit 1 (BOOST TREATMENT GROUP ONLY)**				X							
Shared Reading Video 3					X						
**Visit 2 (BOOST TREATMENT GROUP ONLY)**						X					
Shared Reading Video 4							X				
**Visit 3 (BOOST TREATMENT GROUP ONLY)**								X			
Shared Reading Video 5									X		
**Visit 4: Final Assessment**											X
Expressive One-Word Picture Vocabulary Test-4											X
Receptive One-Word Picture Vocabulary Test-4											X
MacArthur Bates Communicative Development Inventory CAT											X
Video Recorded Speech Task											X
Video Recorded Child Language Sample											X
Shared Reading Frequency Survey											X

Our primary outcomes are children’s expressive and receptive language. Based on studies of children without orofacial clefts, DBS interventions result in moderate improvement in both domains, usually measured using single-word expressive and receptive vocabulary measures [13]. We have incorporated multiple assessment methods for these outcomes, including standardized clinician-administered measures, parent report measures, and observational coding of naturalistic language samples. Staff completing all outcomes assessments will be unaware of children’s randomization. Project staff completing standardized measures will be asked to practice and record an assessment with a non-study child in the target age range. These administrations will be reviewed by one of the investigators (AJ) and scored for reliability before certifying examiners to test RCT participants. During the study, we will video record every tenth administration for reliability scoring. Similarly, staff completing observational coding will practice on sample videos with scores compared to a "gold standard" (LS) to ensure reliability before coding videos for RCT participants. During the study, at least 20% of observations will be coded by more than one observer to assess inter-rater reliability.

#### Clinician-administered measures

Clinician-administered measures will include the Expressive One-Word Picture Vocabulary Test—Fourth Edition (EOWPVT-4) and Receptive One-Word Picture Vocabulary Test—Fourth Edition (ROWPVT-4) for English-speaking families [24]. Both measures have large normative samples, including children as young as two years. For bilingual and Spanish-speaking children, the Spanish-bilingual editions will be used (EOWPVT-4:SBE and ROWPVT-4:SBE) [25, 26]. These measures were normed with a sample matched to the U.S. Latino/Hispanic population based on the 2010 U.S. Census. Bilingual participants are allowed to provide answers in English and Spanish. Correct responses in either language are credited for the total score, and the percentage of correct answers in each language is also calculated. The English and Spanish-Bilingual Editions of the EOWPVT-4 and ROWPVT-4 take about 20 minutes to administer. Reliability is excellent (i.e., internal consistency reliabilities > 0.94, test-retest reliabilities > 0.91), and both scales correlate with other language measures and differentiate children with and without language delays. Both measures have been used in several prior studies documenting the efficacy of DBS and similar interventions [13]. The choice of language for test administration will be based on parents’ reports of the child’s language exposure and production at home.

#### Caregiver report measures

Caregivers’ reports of children’s expressive vocabulary will be measured using the MacArthur Bates Communicative Development Index—Computer Adaptive Test (CDI-CAT) in their first and final visits [27]. The CDI-CAT is normed for ages 15 to 36 months based on 25–50 questions in English or Mexican Spanish and takes about 5 minutes to complete. The CDI-CAT was developed based on 7,633 English-speaking children 12–36 months and 1,692 Spanish-speaking children 12–30 months based on three Communication Development Inventories (CDI) and found the item response theory models had a correlation of 0.92 with the full CDI forms [28].

#### Observational measures

In addition to clinician-administered measures, we will include a parent-child interaction observation using structured tasks to elicit a naturalistic child language sample [29–35]. These interactions will be recorded, transcribed, and coded to assess the number of clear and intelligible utterances, unique words used, and mean length of utterance (MLU). Transcription staff will transcribe at least 10 practice recordings before reviewing study videos. A “gold standard” staff person will independently review at least 20% of the video-recordings after that to monitor for drift. Similarly, before coding transcripts, staff in training will code practice transcripts to establish reliability. After that, two staff will independently code 20% of the transcripts to monitor inter-rater reliability.

### Secondary outcome measure

#### Speech production accuracy and sound diversity

Speech sound production skills are often a concern for children with CP±L, particularly in early childhood. Although data on the effects of DBS interventions on speech are lacking, studies by Scherer et al. suggest benefits for caregiver-focused interventions and scaffolded practice [10–12]. Further, we hypothesize that differences in speech sound production at baseline may influence caregivers’ responses to child vocalizations. For example, some of the core skills in DBS (e.g., reflecting and expanding on the child’s language) may be more challenging when a child has a speech sound disorder. We, therefore, plan to assess speech production skills at baseline and again at the end of treatment. We will use a structured picture naming task developed for this study using around 25 images that children either name or repeat in English, Spanish, or both. The single-word stimuli were selected based on the consonants expected in a child’s sound inventory for each language for ages 24–40 months [36]. Each consonant phoneme will be sampled in at least two positions of stimulus words (initial and final for English, initial and medial for Spanish) to allow for computation of percent consonants correct-revised scores (PCC-R) and to derive a total number of unique consonants produced, serving as an estimated consonant inventory [37, 38]. Speech samples will be video recorded and later transcribed by two speech-language pathologists (SLP) with extensive experience evaluating speech in children with cleft palate. A bilingual SLP will code Spanish-speaking and Spanish-English bilingual children’s samples. We will re-code 20% of samples for intra-rater and inter-rater reliability. Samples will be analyzed in batches, including a random assortment of videos from pre- and post-treatment to minimize bias.

### Intermediate and exploratory outcomes

Throughout the study, we will use observational coding of parents’ book-sharing videos to assess the quality and frequency of book-sharing interactions in the BOOST and SOC groups. These are proximal targets for the intervention, hypothesized to mediate changes in children’s language outcomes. We also plan to code children’s language during book-sharing interactions, exploring whether intervention changes children’s vocalizations (e.g., number of unique words used, mean length of utterance) during reading.

#### Quality of book-sharing interactions

The quality of parent-child interactions during book sharing will be measured by observational coding of participants’ videos at baseline and throughout the intervention. We plan to use an observational coding system based on previous DBS studies and studies of parent-child interactions. The coding reflects observation characteristics (e.g., length, audio quality of video) and parents’ use of DBS strategies, sensitivity, and engagement. Items are rated on 30-second intervals, indicating whether each behavior occurred during the interval. An overall score will be generated, with higher scores indicating better engagement and use of DBS skills.

#### Frequency of book-sharing interactions

The frequency of book-sharing interactions will be tracked via parent reports. We will use an ecological momentary assessment (EMA) approach, sending surveys on a randomly selected five days each week and asking parents (1) whether they read to their child in the last 24 hours, (2) how many books they read, and (3) the book titles. We will generate an average score for each participant, reflecting how many books the parent reports reading to their child daily.

#### Child language during book-sharing

We will transcribe book-sharing interactions using the video recordings shared by parents throughout the study. Child vocalizations will be coded for the number of clear and intelligible utterances, the number of unique words used, and the mean length of utterance (MLU).

### Sample size considerations and participant recruitment and retention

We determined the sample size (N = 320) based on the number of participants needed to ensure 80% power to test the hypothesis that children in the BOOST intervention group will have better receptive and expressive language skills than those in the SOC comparison group. Power analyses were based on two-sample unequal-variance t-tests, an alpha = 0.05/2 to adjust for multiple comparisons, and up to 20% attrition (i.e., final N~256). Under these assumptions, we will have 80% power to detect treatment group differences of >0.4 SD for our primary outcomes. Treatment effects this large have been observed in DBS studies with other non-cleft populations and are considered plausible and clinically meaningful [13].

Our assessment of longitudinal changes in child language during book-sharing interactions (Hypothesis 1b) will use a generalized estimating equations (GEE) approach to linear regression with robust standard error estimates. The outcome for these analyses will be observed child language, calculated as an average number of utterances, unique words used, and mean length of utterance. Analyses will test the effects of the intervention on children’s language during book-sharing interactions over the five observations. We will have > 80% power to detect changes with an effect size > 0.4 between any two time points and changes with effect sizes > 0.3 overall. Meta-analyses of similar interventions with non-cleft participants suggest that effect sizes this large or larger are observed and considered clinically meaningful. Similarly, we will have > 80% power to assess longitudinal changes in children’s language during book-sharing interactions, the quality and frequency of book-sharing interactions, and changes in parents’ book-sharing behaviors as a mediator of differences in children’s language outcomes.

### Recruitment and retention

Collectively, participating centers see approximately 200 new patients with isolated clefts each year. We anticipate a 50% consent rate, allowing us to enroll our target sample with up to 4-years of recruitment. Sites will complete ongoing reviews of medical records to identify potentially eligible participants in our target age range. Study flyers will be made available in the clinic, and information will be shared among cleft team providers, such as nurses, speech-language pathologists, psychologists, social workers, pediatricians, geneticists, and surgeons, so they can also inform families about possible participation. We will approach potentially eligible participants in the clinic or by phone when children are 24 to 32 months old. Parents who express interest will complete a brief interview, in person or by telephone, to confirm eligibility. In addition to receiving children’s books in their preferred language, families will receive incentives for completing study activities for sending shared reading videos ($40 per video shared) and completing the final evaluation ($150). We will also provide the results of children’s end-of-study language assessments in a brief report and encourage parents to share the results with their child’s pediatrician and other providers.

Study visits will be scheduled at times convenient for participating families to facilitate retention. We will use multiple strategies to communicate with families, including text and e-mail reminders for study visits. Participants will be considered "lost to follow-up" if they cannot be contacted, do not respond to outreach attempts, or cannot schedule their child’s outcome assessment within six months of the end of the intervention or active participation (controls). To minimize attrition, we will ask families for the name and contact information of an alternate contact who could help us locate them. We will also use formal and informal search strategies (e.g., online white page searches, Omnitrace) to find families as needed. We will make at least four attempts to contact families using several modes of contact, such as phone, letter, text, and e-mail. Our sample size of N = 320 provides adequate power to address the study aims with 20% attrition.

### Randomization

Half the participants will be randomly assigned to the BOOST Intervention Group (n = 160). The other half of the participants (n = 160) will be randomized to a SOC comparison group that receives the same children’s books, uploads shared reading videos, and responds to surveys but does not receive individualized BOOST intervention sessions. Randomization will be stratified by study site, families’ preferred language (English or Spanish), and Medicaid status. Randomization will be blocked in blocks of 4 to 6 participants to ensure reasonable balance in small strata.

A data manager will prepare randomization lists for all sites using REDCap under the direction of the study biostatistician. These lists will be concealed from all other study staff. Once a patient has been determined eligible for participation and randomization, the site coordinator will enter the participant into the study database and receive the treatment assignment via REDCap.

Staff completing outcomes assessments (e.g., observational coding of parent-child interactions, child language assessments) will be unaware of group assignments. Masking will not be feasible for some procedures that, by definition, reveal participants’ group assignment (e.g., observational measures of treatment fidelity). We will use restrictions on user privileges in REDCap to limit access to this information in project databases, and we will use password/code protections to restrict access to calendars, data archives used to store videos, and any other information that might reveal group randomization. Masked staff will not participate in meetings where group assignments may be revealed. If an assessor or observational coder becomes aware of a participant’s group assignment, we will identify another staff member to complete the assessment/coding.

### Data management

We plan to use direct entry into centralized REDCap databases to reduce the use of paper record forms and data entry steps. Before enrolling participants, staff involved in data entry will pilot test databases with sample record forms to ensure accuracy and assess any database issues that need to be addressed. Regular study coordinator teleconferences will address questions throughout the study and ensure continuity across centers. All data entry forms and databases will be password protected, with access restricted depending on staff roles/responsibilities. The study team and data entry staff will ensure that data entry is accurate and complete. Seattle Children’s Research Institute staff will run monthly reports to identify missing data with logic checks to assess potential data entry errors. The investigator or designee will review unanticipated problems and adverse events.

### Statistical methods

In analyses for our primary outcomes, we will use an intention-to-treat (ITT) approach to test the hypothesis that children in the BOOST group exhibit better language skills than children in the SCO comparison group. We will calculate 2-sample unequal-variance t-tests to estimate treatment versus control group differences in child language outcomes post-intervention. We will calculate standardized treatment effect sizes (ES) for all analyses to allow comparison across outcomes with different measurement scales. We will not adjust for covariates in the primary analyses. However, in sensitivity analyses, we will use bias-corrected ITT analyses to examine the robustness of treatment effects in the setting of non-adherence, missing data, or study attrition. To assess attrition bias, we will explore outcomes using Inverse Probability Weighting (IPW), with weights determined by the probability of having missing data or being lost to follow-up based on the RCT stratification variables and demographic and clinical data [39]. We will also conduct sensitivity analyses to examine the effects of variation in treatment fidelity using a weighting approach. Finally, we will examine the potential effects of children’s participation in other interventions in the community (e.g., speech and language therapy) using censored normal regression (CNR) [40].

Longitudinal analyses examining changes in child language during book-sharing and caregiver behaviors (i.e., quality and frequency of book-sharing) will use a GEE approach to linear regression with robust standard error estimates. This approach accounts for the correlation of observations over time and allows for the use of partial data on subjects who are lost to follow-up or non-compliant. Estimates will be adjusted for the RCT stratification variables, demographic confounds, and clinical characteristics (e.g., cleft type, age at cleft repair). Smoothing splines will be fit to examine changes in parents’ reading behaviors over time, including non-linear trends.

We will use a multi-step linear regression approach to examine whether changes in parents’ reading behaviors mediate differences in children’s language outcomes. Mediators include: (1) shared reading frequency, (2) overall quality of parent-child interactions, and (3) parents’ shared reading skills, which will be assessed throughout the intervention period. Mediators will be analyzed separately to determine their individual effect on child outcomes and to avoid a washout effect from including all mediators in a single model. We will not adjust for multiple comparisons for this aim. Each regression model will follow the same basic approach to analyzing the effects of adjustment for a given mediator (Z) on the association between the intervention (X) on the child language outcome (Y). We will run two models per outcome: (1) Y = α + βX + e1, and (2) Y = A + BX + CZ + e2. The terms e1 and e2 represent model errors. Model 1 describes the unadjusted effect of the intervention on the outcome. Model 2 illustrates the combined effect of intervention and mediator on outcome. A comparison of the coefficients β and B indicates the degree of mediation. For example, β = B >0 suggests a positive intervention effect that is not mediated by Z (i.e., the null hypothesis of no mediation), whereas β >0 and B = 0 reflects a positive effect fully mediated by Z. Intermediate values reflect the degree of mediation (e.g., B = β/2 represents 50% mediation). Confidence intervals for the degree of mediation will be obtained using the bootstrap methods.

Finally, we will explore demographic and clinical characteristics associated with "good" versus "poor" treatment responses. In these analyses, we will examine parent reading behaviors and child outcomes in relation to clinical and demographic characteristics (e.g., language spoken in the home, family composition, parents’ education level, the child’s cleft type, child’s hearing status, sex of the child, study center/interventionists). We will use a two-step process for model selection and analysis. First, we will identify children who score > 0.5 SD above or below the mean relative to their intervention group. Second, we will examine the distribution of each characteristic of interest and identify those that (a) differ in distribution between those who score above-expected values, below-expected values, and as-expected and (b) have sufficient cell sizes (n >30). Selected variables will then be included in multivariate linear regression models to evaluate whether parents’ reading behaviors and child language outcomes vary across subgroups. We will calculate correlations between each variable before fitting any model, combining or excluding variables to avoid multicollinearity. To prevent overfitting, no more than 12 terms will be included in the model.

### Study monitoring, ethical considerations, and dissemination

#### Study monitoring

This is a low-risk study, and a data monitoring committee was not considered necessary. We do not plan interim analyses of the primary, secondary, or exploratory outcomes. Interim analyses may be used for quality assurance, including to ensure inter-rater reliability in observational coding and speech assessments and to analyze missing data.

### Ethical considerations and consent/parent permission

A possible breach of confidentiality is a particular concern for our study, given the use of in-home video recordings and study visits. We have taken several steps to protect confidentiality. In our video recording instructions for participating parents, we ask that they ensure that recordings do not capture anyone else in the home. We ask that parents record in a private setting and inform others in the house when they begin recording. We developed an app for data collection that allows caregivers to review all videos before sharing them with our team. Videos are securely shared to a file that is only accessible to research team members and saved in a password-protected file. Parent-child book-sharing videos will be coded and retained until child participants turn 18. Any future use would require separate IRB approval. Once participants turn 18, we will permanently delete recordings. Study visits are conducted using a HIPAA-compliant platform (Zoom). Sessions are video recorded for review of treatment fidelity, and videos will be permanently deleted once the recordings are coded. Other steps to protect confidentiality include using study identification numbers instead of identifying information. Only certain study team members can access the "link" between these study identification numbers and participant identifiers. During consent, we inform parents of the potential limits to confidentiality (e.g., instances of suspected abuse or neglect).

We established single IRB reliance for all sites, with Seattle Children’s being the primary IRB for the study. The Seattle Children’s IRB approved the protocol, informed consent form(s), recruitment materials, and all participant materials on November 7, 2024 (IRB# 00004565). Other participating centers are were activated on May 10, 2024 (Nationwide Children’s Hospital) and May 16, 2024 (Children’s Hospital Los Angeles). An IRB-approved consent and parent permission form describing the study procedures and risks will be sent or given to participants in their preferred language (English or Spanish). Our team includes multiple bilingual members, and we will ensure that a team member fluent in Spanish is available to review the consent and answer any questions for Spanish-speaking families. Before completing any study-related procedures, we will document consent in writing or using e-consent.

Per NIH guidelines, we will make de-identified data available to other research groups with appropriate IRB approvals, and results will be submitted to ClinicalTrials.gov. We have established an analysis and dissemination plan for our team members who are interested in new analyses. This includes generating a brief (1-page) proposal to be reviewed by the PI and site PIs to assess the scientific merit of the analysis proposal and any potential conflicts with other work in progress or planned analyses. Once approved, analysis proposals will be sent to all investigators to invite participation as co-authors on the writing team. Site PIs can nominate other trainees, staff, or collaborators who can be invited to participate in dissemination activities. We will provide potential co-authors with guidelines from the International Committee of Medical Journal Editors (ICMJE) and ask for confirmation that they meet the criteria for co-authorship. Authorship order will be determined based on contribution [41]. We will prioritize opportunities for new investigators and trainees to serve as first authors and site-PIs to serve as senior/last authors.

## Results

The BOOST study protocol has been approved by NIH/NIDCR (Protocol #23-048-E) and registered with Clinicaltrials.gov (NCT06338319). Two bilingual interventionists (CT, CC), who were both involved in developing the BOOST program, are prepared to begin intervention sessions. We will hire and train 1–2 additional interventionists in the coming months. Staff have been employed to complete observational coding and outcomes assessments with ongoing training. We have enrolled two pilot participants for a "wet run" of the intervention and data collection procedures. We began enrolling participants for the RCT in April 2024.

## Discussion

This will be the first large, multi-center study of an intervention to promote language development in children with CP±L, with significant potential to increase understanding of the malleability of deficits observed in previous studies and improve the care standard for affected children. Our population will be slightly younger at enrollment (i.e., ages 24 to 32 months) than many other DBS studies. We chose this age range to correspond with typical ages for cleft treatment in tertiary care centers. For example, palate surgeries are typically completed by 12 to 18 months, and children are usually seen for regular (annual) multi-disciplinary evaluations, including speech assessments, by 24 months. Developmentally, this corresponds with rapid increases in children’s vocabulary and spoken language, making this an important time to optimize children’s language environment. Finally, including English and Spanish-speaking participants will allow us to enroll a larger proportion of the patients in participation centers.

### Limitations

Anticipated limitations and potential pitfalls for our study include limited generalizability to patients with clefts in conjunction with known genetic syndromes and other malformations. These groups are known to be at greater risk for poor neurodevelopmental outcomes, and we anticipated that their needs and response to intervention would likely differ. It is also possible that some patients will receive an exclusionary diagnosis (e.g., ASD) after study completion since these conditions may not be identified by 24- to 32-months. Developing similar interventions for children with syndromic conditions is a high priority. However, in this initial study, a more clinically homogeneous patient group was needed to establish intervention efficacy. Including English and Spanish-speaking participants complicates our outcomes assessment, given potential differences in language development for multi-lingual children. We have accounted for this by including language as a stratification variable in randomization, helping to ensure that the groups are balanced. The intensity of our intervention is another consideration, and we acknowledge that three 50-minute visits are modest. The results of meta-analyses have been somewhat contradictory on the minimum duration needed for similar interventions, with some indication that interventions of 90 minutes or greater produce significant treatment gains and others suggesting that longer duration is necessary for populations at significant risk for language delays [13, 14]. Ultimately, a lower-intensity intervention was needed to deliver the program at scale while minimizing participant burden and maximizing subsequent adaptation into standard cleft care. Finally, given the young age of our participants, there is a risk that our outcome measures will not be sensitive enough to detect treatment gains. Our multi-method assessment, including caregiver reports, clinician-administered measures, and observational measures of outcomes proximal to the intervention (e.g., changes in caregivers’ reading behaviors and changes in observed child language), is intended to address this concern.

### Conclusion

The BOOST study will contribute to the limited literature on parent-focused interventions to promote language development in children with CP±L. The study will also advance knowledge of remote intervention delivery, with applications for geographically dispersed patient populations that would be difficult to serve with in-person developmental interventions.
